# Optimization by Self-Organized Criticality

**DOI:** 10.1038/s41598-018-20275-7

**Published:** 2018-02-05

**Authors:** Heiko Hoffmann, David W. Payton

**Affiliations:** HRL Laboratories, LLC, 3011 Malibu Canyon Rd, Malibu, CA 90265 USA

## Abstract

Self-organized criticality (SOC) is a phenomenon observed in certain complex systems of multiple interacting components, e.g., neural networks, forest fires, and power grids, that produce power-law distributed avalanche sizes. Here, we report the surprising result that the avalanches from an SOC process can be used to solve non-convex optimization problems. To generate avalanches, we use the Abelian sandpile model on a graph that mirrors the graph of the optimization problem. For optimization, we map the avalanche areas onto search patterns for optimization, while the SOC process receives no feedback from the optimization itself. The resulting method can be applied without parameter tuning to a wide range of optimization problems, as demonstrated on three problems: finding the ground-state of an Ising spin glass, graph coloring, and image segmentation. We find that SOC search is more efficient compared to other random search methods, including simulated annealing, and unlike annealing, it is parameter free, thereby eliminating the time-consuming requirement to tune an annealing temperature schedule.

## Introduction

Evidence suggests that the healthy mammalian brain operates in a critical state^1–4^. Critical systems are dynamical systems with many interacting particles or components that display scale-invariant fluctuations. Scale invariance has long fascinated the human mind. The laws of physics are not invariant to scale: a 10-times wider and longer wooden cylindrical beam cannot support a 1000-times larger weight. Yet, in nature, we find many examples of approximate scale-invariance, e.g., the fractal shape of flowers and trees with repeating structures at different scales.

In physics, we usually achieve criticality by fine-tuning a control parameter, e.g., the temperature, to a specific value at which we observe the scale-invariant fluctuations (see, e.g., the liquid-vapor critical point). Yet, in many natural systems, criticality occurs even without such fine-tuning, and, apparently, the brain is one example of such a system. This self-tuning to criticality has been called self-organized criticality (SOC)^5^.

Criticality has been further referred to as a special state between order and chaos^2^: systems are ordered at low temperatures and random at high temperatures, but appear the most complex at the critical temperature. So, it is appealing to think that the brain operates at criticality. Interestingly, experiments show that when the brain malfunctions, e.g., during epileptic seizures, the brain loses the characteristics of criticality^4^.

In the brain, criticality is often characterized by neural avalanches. Neural avalanches are bursts of neural activity, e.g., as recorded with multi-electrode arrays measuring local field potentials (LFPs)^1^. Here, synchronous LFP spikes across multiple electrode locations constitute an avalanche. Experiments show that the sizes of neural avalanches follow power-law distributions^1^, which are generally a consequence of scale invariance and thus evidence for criticality, and neural models have been developed that automatically tune networks towards the critical state^6^.

It is still debated, however, if the brain is actually in a critical state and what the benefits of such a property are^3,7,8^. As benefits, optimal dynamic range, memory, and computational power have been suggested^2,3,9^. For example, Bertschinger and Natschläger^10^ show that recurrent neural networks perform best at the critical state; there, however, the task was simple (3-bit parity), and a tuning parameter was required to achieve the critical state.

In neural network models, the ability to reach an SOC state is typically studied in isolation, without having the network at the same time carry out a “useful” computational task (see, e.g., Stepp *et al.*^11^). On the other hand, the useful deep-learning networks are not critical. The difficulty to design a network that is critical and useful at the same time motivated this present work: we isolate the SOC dynamics in one network and then use these dynamics to drive a separate system. So, we create a novel optimization algorithm that uses the SOC process to create test patterns for optimization.

To solve an optimization problem, we initially identify a graph of linked/coupled optimization variables (which, e.g., are linked through an equation). Then, we create a new graph for the SOC process that mirrors the optimization graph such that there is a one-to-one correspondence between the optimization variables and the nodes in the SOC graph. The SOC process will continuously create avalanches on its graph, and the correspondence allows us to map these avalanches onto test patterns of the optimization variables.

Surprisingly, this method, which we call SOC search, can efficiently find approximate solutions to non-convex optimization problems that have many local minima. We demonstrate the performance of SOC search on a variety of optimization problems and compare against simulated annealing^12^ and random search. In contrast to simulated annealing, SOC search needs no parameter tuning so long the graph structure on which the SOC process is computed matches the optimization problem.

The reminder of this article is organized as follows. Section 2 introduces a popular model of SOC, the Abelian sandpile model^5^, which is also the basis for our optimization process. Section 3 describes the SOC optimization algorithm. Section 4 describes our experiments, introducing each optimization problem: Ising spin glass, graph coloring, and image segmentation; the latter optimizes the energy of a Markov random field. Finally, Section 5 discusses the benefits and limits of SOC search and summarizes the work.

## Self-Organized Criticality

Self-organized criticality has often been illustrated with a sand pile^5,13^. Grains of sand are added one at a time, and eventually, the slope of the pile converges to a specific angle. At this angle, small and large avalanches occur, and the system is balanced just right such that the avalanche sizes follow a power law (this example is illustrative; not all real sand piles are actually self-organized critical^13^). The system self-organizes because the slope self-adapts: grains are added to a shallow slope until avalanches occur, and a slope too steep will collapse in a large avalanche.

Bak *et al*. developed a model that mimics the sandpile dynamics, and this model is actually self-organized critical^5^. This model, called Bak-Tang-Wiesenfeld model or Abelian sandpile model^14^, has been originally defined on square and cubic lattices, but it can be generalized to arbitrary graphs^14^. Here, we consider this generalized version.

Assume a graph of nodes *i* that each contain *x*_*i*_ amount of grains. The Abelian sandpile model consists of a slow and a fast process. The slow process adds one grain to a random node *i*, *x*_*i*_ → *x*_*i*_ + 1 (slow external driving force). The fast process computes the propagation of an avalanche, as described in the following. If a node is above threshold *x*_*i*_ > *d*_*i*_, where *d*_*i*_ is the degree of node *i*, then it sheds one grain to each of its *d*_*i*_ neighbors,

1 $${x}_{i}(t+\mathrm{1)}={x}_{i}(t)-{d}_{i}$$

2 $$\forall j\in {{\mathscr{N}}}_{i}\,:{x}_{j}(t+\mathrm{1)}={x}_{j}(t)+1,$$

where ${{\mathscr{N}}}_{i}$ is the set of nodes that share an edge with node *i* (here, we consider only undirected graphs). This shedding may result in a neighbor to be above threshold, making this neighbor to also shed its grains according to the above equations. This repeated shedding can result in a cascade of events, an avalanche of grain topplings. Once an avalanche is complete, i.e., all nodes are at or below threshold, the slow process proceeds by adding another grain.

**Figure 1:**
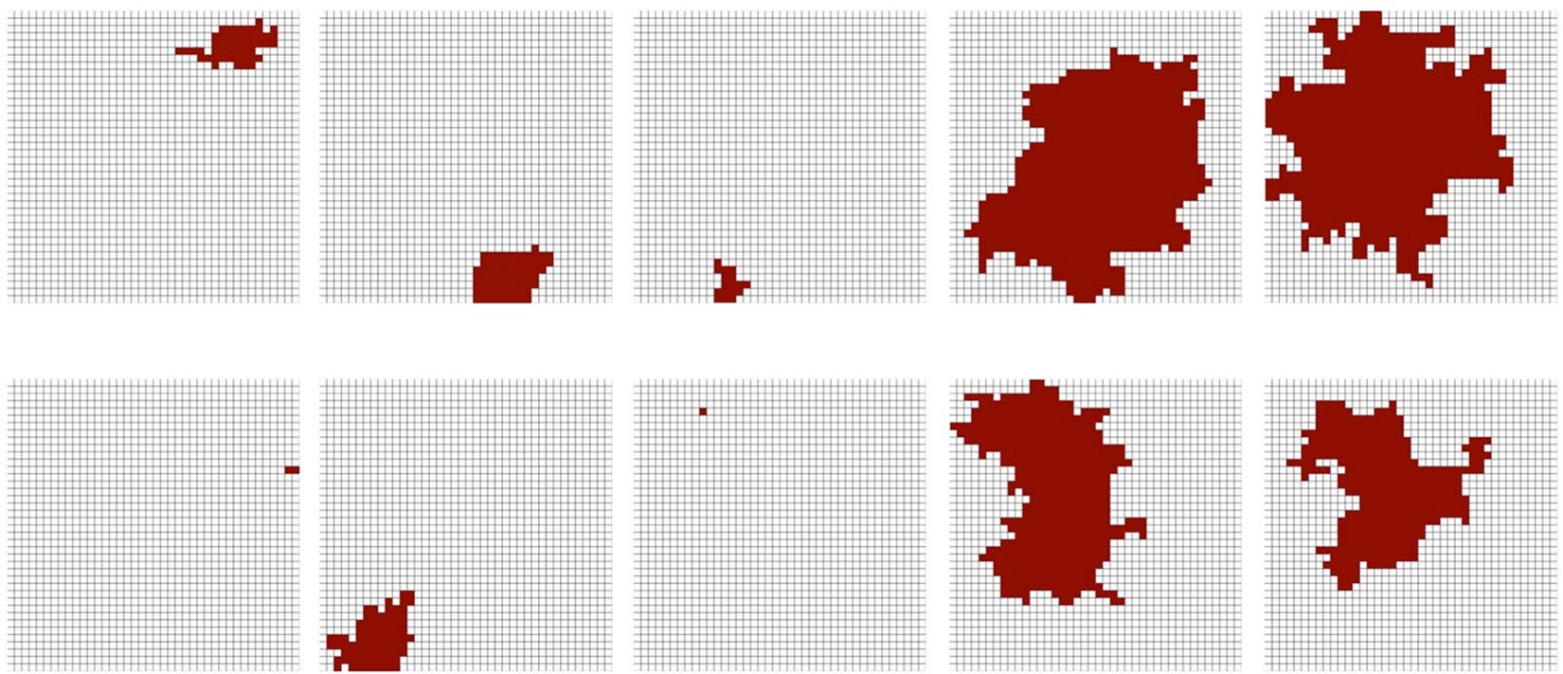
Sample sequence of avalanches from an SOC model on a 40 × 40 square lattice.

After an initial phase of self-organization, avalanches occur with sizes that follow a power law distribution (this distribution has an exponential cutoff because the number of nodes is limited). For a square lattice, Fig. 1 shows a typical sequence of avalanches. They occur at different locations, have different sizes, and appear to have fractal-like boundaries.

When we continuously add grains, they also have to disappear eventually. In the original model, the square lattice has edges and the grains just fall over the edges and disappear. In the generalized version, we need to add either a sink node or dissipation. All grains that arrive at a sink node disappear. With dissipation, grains disappear with a small probability when moving between nodes. Strictly, this dissipation breaks the SOC characteristics, but if it is sufficiently small it is not noticeable, because the exponential cutoff is dominated by the limited system size. When we increase the system size, we also need to reduce the dissipation accordingly to eliminate its impact. In our experiments, we used either a square lattice with edges or random graphs with dissipation (with a probability of 0.05).

## SOC for Optimization

We use the above SOC process for non-convex optimization. To do so, we need an optimization problem with a graph structure; this article contains three examples. For example, in graph coloring, the optimization variables are the color values at each node of a graph. In an Ising spin glass, the optimization variables are the orientations of the spins that are energetically coupled to their neighbors. Moreover, we assume that the optimization variables are discrete, ∈{*a*_1_, ..., *a*_*k*_} with *k* different values.

Initially, we copy the graph structure, the set of vertices and edges, of the optimization problem and compute on this graph the Abelian sandpile model. Thus, there is a one-to-one correspondence between the nodes of the sandpile model and the optimization variables. In the sandpile model, the nodes are initialized with random grain numbers drawn uniformly from {1, ..., *d*_*i*_} for each node *i*. Grains are added and avalanches are computed until the SOC state is reached. Then, the actual optimization process begins (Algorithm 1).

**Algorithm 1:**
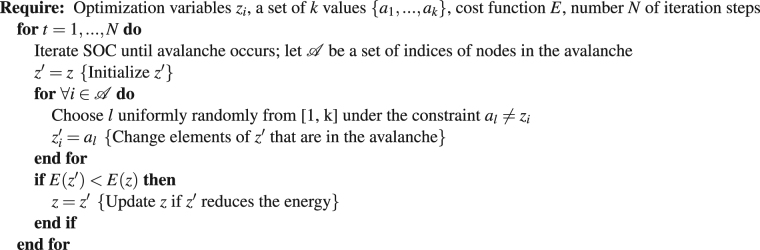
SOC Search.

The avalanches act as delta patterns that change the corresponding optimization variables, computing test patterns *z*′. For *k* = 2, this change simply flips the variables (e.g., spins) in a test pattern. The resulting test patterns are used in a greedy optimization scheme. This process is iterated for a fixed number of iteration steps or until a stop criterion is reached, e.g, the change in energy *E* is below a threshold. Interestingly, the variables *x*_*i*_ in Equations (1) and (2) are independent of the optimization variables *z*; there is no feedback onto the SOC process.

Other work before has combined the concepts of self-organized criticality and optimization, see extremal optimization^15^. Different from ours, however, that work uses an optimization algorithm *inspired* by SOC, but uses neither an SOC process to generate delta or test patterns nor SOC on a graph.

## Results

We chose three optimization problems to demonstrate the functionality and versatility of SOC search. Apart from copying the graph structure, no adjustment was required for each problem. The following three sections show results and describe our experiments for 1) optimizing the ground state of an Ising spin glass, 2) graph coloring, and 3) image segmentation.

### Ising Spin Glass

We tested SOC search for finding the ground state of an Ising spin glass, which is a common benchmark test for optimization methods - see, e.g., Santoro *et al.*^16^. An Ising spin glass is an arrangement of spins, particles with a magnetic moment, in a lattice. The spins, *s*_*i*_, point either up or down and are coupled to their neighbors such that they prefer to have either aligned or opposing magnetic movements, depending on a coupling matrix, *J*_*ij*_. In the ground state, i.e., without any thermal fluctuations, the spins orient themselves to minimize the energy

3 $$E=\sum _{i,j\in {{\mathscr{N}}}_{i}}{J}_{ij}{s}_{i}{s}_{j}\mathrm{.}$$

**Figure 2:**
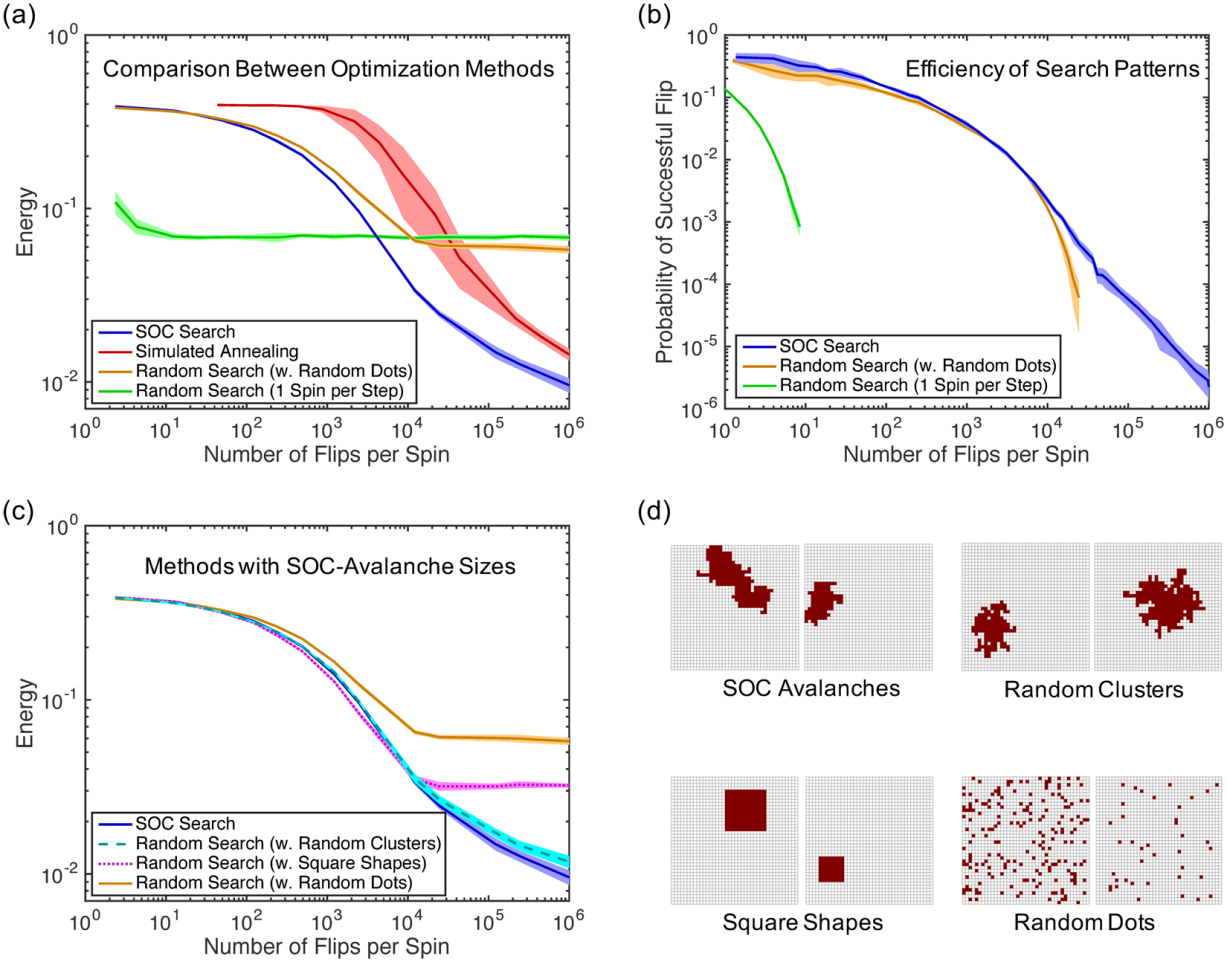
Results for optimizing the energy of an Ising spin glass on a 80 × 80 lattice (**a**,**c**) and probability of improving the energy in a greedy search (**b**), showing mean ± std (*n* = 10). Search-pattern samples are shown in (**d**). In (**b**), the results for Random Clusters and Square Shapes have been omitted for clarity because Random Clusters produced similar probabilities as SOC Search and Square Shapes similar probabilities as Random Dots.

The spins can have two states $\frac{1}{2}$ or $-\frac{1}{2}$. Here, we chose a 80 × 80 lattice, and the *J*_*ij*_ were chosen randomly uniformly from the interval (−2; 2). On such a lattice, polynomial algorithms exist to find the ground state (on generic graphs the problem is NP hard); still for approximate solvers like ours, the difficulty is in principle the same, and therefore, the lattice is suitable for benchmarking^16^. We chose the square lattice since methods exist to compute the exact energy minimum, which we obtained from the Spin Glass Server^17^ and subtracted from Equation (3); so, zero energy is the optimum (Fig. 2).

We compared SOC search against three methods: random search flipping one spin, random search flipping as many spins as the size of an SOC avalanche, and simulated annealing^12^. The first two serve as baselines and the third to illustrate the competitiveness of SOC search. One-spin random search is doing a greedy optimization flipping one random spin at a time.

To demonstrate that the shape of the SOC avalanche matters and not just the size distribution, we split the random search with avalanche sizes into three variants: random dots, square shapes, and random clusters. For each, the operation is similar to Algorithm 1, but instead of using the avalanche nodes ${\mathscr{A}}$, $|{\mathscr{A}}|$ nodes are chosen randomly according to one of the following three procedures: 1) *Random dots:* A set of $|{\mathscr{A}}|$ dots/nodes is uniformly randomly chosen from the spin lattice. 2) *Square shapes:* A square with an area matching $|{\mathscr{A}}|$ as close as possible is placed at a random location (uniformly distributed). 3) *Random clusters:* A random cluster is grown starting from a uniformly randomly placed seed location. The cluster grows one spin at a time until it reaches the corresponding SOC-avalanche size. In each growth step, one spin is placed at a random location uniformly distributed among all sites that neighbor spins in the current cluster. These random clusters were most similar to the SOC patterns compared to the other random pattern variants (Fig. 2d).

Simulated annealing^12^ is often the method of choice when dealing with problems that have many local minima. The annealing process helps escape local minima by allowing moves to states with higher energy. These moves happen with probability *p* = exp(−Δ*E*/*T*), where *T* is a temperature-like variable. At high temperature, moves to a higher energy are likely, but when the temperature approaches zero, the algorithm becomes greedy. Theoretically, simulated annealing is guaranteed to find a global optimal, but only in infinite time. In practice, simulated annealing depends on the proper choice of the initial temperature and temperature schedule, also called annealing schedule.

Here, we used the same annealing schedule as in Santoro *et al*.^16^, a linearly decreasing temperature with a starting value of *T* = 3. Like for one-spin random search, we tested the energy change of flipping one spin at a time and flipped the spin according to above probability. Different from random search, we did not choose the spins randomly, but chose the nodes in the lattice in sequence and repeated the sequence over and over again. This strategy produced better results than the random choice.

For a fair comparison of the computational cost, we count the number of spin flips for each method because the cost of evaluating a change in energy is linear in the number of nodes flipped. For SOC search, we flip all spins in an avalanche; so, we require more flips per iteration than simulated annealing. This evaluation actually puts our method at a disadvantage because, optimally, we would need to evaluate the energy at only the boundary of an avalanche (if two neighboring spins flip, the contribution of their edge to the energy remains unchanged, see Equation 3). Still, we used this evaluation for simplicity. In the computational cost, we omitted the cost of computing the Abelian sandpile model itself because this process could be either computed in parallel or beforehand: since the sandpile operates independently without feedback, we could compute the avalanches in advance and replay from memory.

As a result of the optimization, SOC search reached the lowest energy values with the fewest number of spin flips (Fig. 2a). Simulated annealing required about 8× more flips for reaching the same energy levels. The random searches with one spin, random dots, and random squares got stuck in local minima. The random clusters performed almost as well as SOC search, but reached significantly worse energy values for sufficiently many iteration steps.

We averaged all results over 10 optimization runs, each starting with a different random spin configuration, with the same starting configuration used across all methods. As in Santoro *et al*.^16^, each data point on the energy curves is from a separate set of 10 simulation runs because simulated annealing requires a unique annealing schedule for each data point. Once annealing reaches zero temperature, it gets stuck in local minima like one-spin random search; so, the annealing schedule has to be adapted for each run.

To gain a greater insight into the workings of SOC search, we computed the probability of energy improvements in the greedy search (Fig. 2b). After many iterations, i.e., 10^4^ flips per spin, the probability to improve the energy with SOC avalanches becomes much larger than with just avalanche-sized random-dot patterns. Instead of dropping off abruptly, the probability decayed more slowly according to a power law, having a slope of −1.5.

### Graph Coloring

Graph coloring has many applications such as scheduling^18^, register allocation^19^, and timetabling. The objective in graph coloring is to color nodes in a graph such that no neighboring nodes are of the same color. On generic graphs, coloring is NP hard. The optimization problem is to find the smallest number of colors possible without edge violations.

To apply simulated annealing and SOC search, we need a suitable cost function. Here, we choose one that has been shown to work with simulated annealing:^20^

4 $$E=-\sum _{i=1}^{k}|{c}_{i}{|}^{2}+\sum _{i=1}^{k}\mathrm{2|}{c}_{i}||{e}_{i}|,$$

where |*c*_*i*_| is the number of nodes in color class *i*, |*e*_*i*_| the number of bad edges attached to nodes of color *i*, and *k* an upper bound on the number of colors. Using this cost function, legal colorings are local minima^20^, and the number of colors is implicitly minimized and is obtained as the number of nonzero |*c*_*i*_| values.

We compared SOC search against one-node random search, random search with random dots, and simulated annealing. For one-node random search and simulated annealing, the search selected one random node at a time. We used an exponentially decaying annealing schedule (similar to Johnson *et al.*^20^), starting at *T* = 10. Preliminary tests found that this schedule worked better for graph coloring.

**Figure 3:**
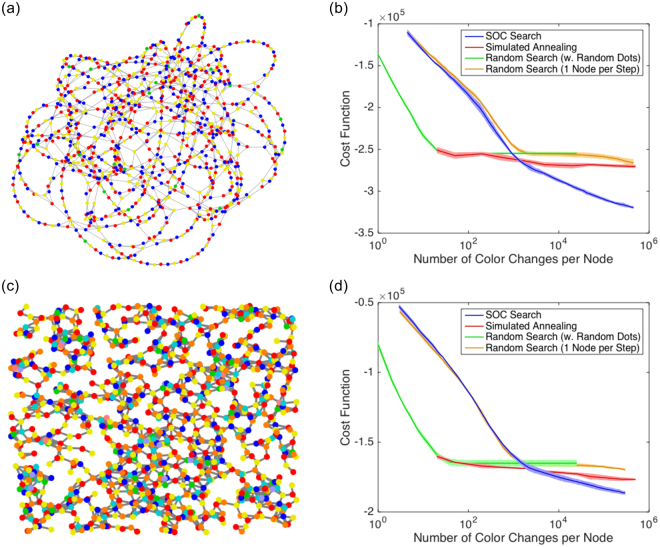
Results of graph coloring on a small-world graph (Top) and a random geometric graph (Bottom), showing a legal coloring produced by SOC search (Left) and cost functions (Right), mean ± std (*n* = 5).

We tested two random graphs: a small world^21^ and a random geometric graph, both with 1,000 nodes (Fig. 3 Left). In the small world graph, each node had 4 neighbors, and the probability of random re-connections was *p* = 0.05. The random geometric graph had an average degree of 6.7. For coloring, we chose *k* = 6 for the first graph and *k* = 10 for the second. Similar to above, we evaluated the computational cost in terms of the number of color changes per node, and as above, for simulated annealing, we re-ran the optimization for each displayed number of color changes.

On both graphs, SOC search produced the smallest cost values (Fig. 3). The results were averaged over 5 optimization runs. For the small-world graph, only SOC and simulated annealing found a legal coloring with 4 colors (other methods needed 5 colors), and for the random geometric graph, only SOC and simulated annealing found a coloring with 9 colors (other methods needed 10 colors).

Surprisingly, despite the random color choice for each node in an avalanche (see Algorithm 1), the path-connectedness of an avalanche still mattered. However, the SOC benefit is smaller the more colors we need to choose from. Simulated annealing does outperform SOC for a graph that requires many different colors (100) and is closer to a fully connected graph, e.g., an Erdos-Renyi graph with 1,000 nodes and probability *p* = 0.5 for connecting two nodes (data not shown).

### Image Segmentation

Image segmentation separates images into distinct regions and is commonly used to separate foreground objects from background. Here, for demonstration, we focus on feature-based image segmentation, which considers local features like color and texture. A different approach is semantic segmentation, which considers object ID - see, e.g., Long *et al.*^22^. Feature-based segmentation is useful for automatic separation of objects from background, e.g., for image editing. We chose image segmentation as our final test because it allows us to visually evaluate the optimization results.

A powerful method for feature-based image segmentation are Markov random fields^23,24^. Using a Markov random field, the set of pixel labels, ${\mathscr{S}}$, is a random field, i.e., each pixel label is modeled as a random variable. The goal is to find a segmentation ${\mathscr{S}}$ that maximizes the posterior $P({\mathscr{S}}|{\mathscr{I}})$ for a given image ${\mathscr{I}}$. The posterior is computed according to Bayes rule, $P({\mathscr{S}}|{\mathscr{I}})\propto P({\mathscr{I}}|{\mathscr{S}})P({\mathscr{S}})$.

The two required factors are the probability for the image model, $P({\mathscr{I}}|{\mathscr{S}})$, and the prior probability of the image labels, $P({\mathscr{S}})$. Here, for the image model, we use a Gaussian distribution as in Kato and Pong^24^,

5 $$P({\mathscr{I}}|{\mathscr{S}})=\prod _{i}\frac{1}{\sqrt{{(2\pi )}^{n}|{{\rm{\Sigma }}}_{{s}_{i}}|}}\exp (-\frac{1}{2}({f}_{i}-{\mu }_{{s}_{i}}){{\rm{\Sigma }}}_{{s}_{i}}^{-1}{({f}_{i}-{\mu }_{{s}_{i}})}^{T}),$$

where *f*_*i*_ is the feature vector for pixel *i*, ${\mu }_{{s}_{i}}$ the feature center for label *s*_*i*_, and ${{\rm{\Sigma }}}_{{s}_{i}}$ the covariance matrix for label *s*_*i*_. For the prior, it is common to use a Gibbs distribution^24,25^,

6 $$P({\mathscr{S}})=\frac{1}{Z}\prod _{i,j}\exp (-{K}_{ij}),$$

here, with *K*_*ij*_ = *γ*exp(−*β*||*f*_*i*_ − *f*_*j*_||^2^) if *i* and *j* are neighbors and belong to different label classes, otherwise *K*_*ij*_ is zero. So, if neighboring features are different, *K*_*ij*_ is approximately zero and does not penalize the probability $P({\mathscr{S}})$, but if the neighbors have similar features even though they belong to different classes, we get a penalty: the prior probability is reduced. Our *K* function is a slightly modified and simplified version of the function used in Kohli and Torr^25^, because our *K* produced better results in our experiments.

Instead of maximizing the posterior directly, it is common to minimize the exponent and define it as an energy^23,24^; so, $P({\mathscr{S}}|{\mathscr{I}})\propto \exp (-E)$ with $E=-ln(P({\mathscr{I}}|{\mathscr{S}}))-ln(P({\mathscr{S}}))$. Numerically, computing with the exponents is more robust. In the following, we use this energy for optimization, setting the constants *γ* = 5.0 and *β* = 1.0.

To obtain the image features, we combined color and local texture values^24^. First, images were converted to LUV color space, and the resulting three colors are the first three dimensions of the feature vector. Then, the LUV images were converted to grayscale and filtered with Gabor and Gauss filters. We repeated the filtering for 4 orientations (0°, 45°, 90°, 135°) of Gabor filters and stored the resulting values after Gaussian smoothing into the feature vector, so each pixel has a 7-dimensional feature vector (3 colors + 4 textures).

**Figure 4:**
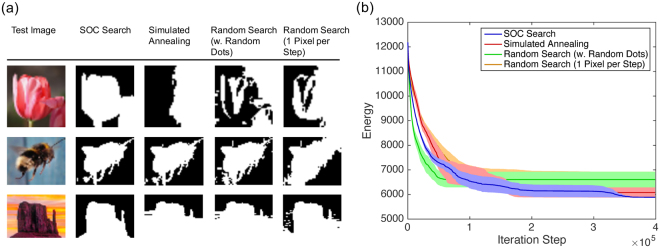
Result of unsupervised image segmentation: worst-case segmentations out of 7 optimization runs (**a**) and energy curves for the tulip segmentation (**b**), mean ± stderr (*n* = 7).

For optimization, we segmented images into two label classes and compared the same four methods as above for graph coloring. SOC search produced the most consistent and meaningful segmentations (Fig. 4). For the energy curves, results were averaged over 7 optimization runs. Here, instead of choosing the number of flips on the x-axis, we used the number of iterations (increments of *t* in Algorithm 1) because $P({\mathscr{S}}|{\mathscr{I}})$ is computationally expensive and has to be re-evalated after each label change. Figure 4a shows the worst-case segmentations over 7 runs for each test image. Here, SOC produced almost the same result in all 7 simulation runs, while the other methods produced occasional bad results. For the best-case segmentations (not shown), the results of SOC search and simulated annealing were the same; the other methods did slightly worse.

## Discussion

For the first time, we used the avalanche patterns of an SOC process to alter test patterns for optimization. Surprisingly, this simple method performs remarkably well on non-convex optimization problems. On the same problems, other greedy methods get stuck in local minima, but SOC search overcomes these minima without using any annealing, without even using any parameter tuning.

A disadvantage of simulated annealing is its dependence on the annealing schedule and parameters. Its performance critically depends on the starting temperature and the number of annealing steps. Moreover, before an optimization problem is solved, it is unknown how many annealing steps are required. This limitation is a big disadvantage because once the temperature reaches near zero, the optimization ends in a local minimum. So, simulated annealing has to be restarted with a new pre-defined number of annealing steps. In contrast, the SOC process is invariant in time (over sufficiently long time scales), and a given solution can be further improved with additional SOC iterations.

Our experiments further demonstrate that the shape of an avalanche matters: the size distribution itself was insufficient for a good optimization performance. With random dot and square patterns, the optimization got stuck in local minima. In our experiments with different shaped patterns, the only difference was the shape; the temporal sequence of sizes was exactly the same between methods since the optimizations were carried out in parallel using the same SOC process to generate the size distribution. The closer the shape of the random patterns resembled the SOC avalanches the better their performance. Our random clusters resembled avalanches and almost reached the performance of SOC, but there are still differences between these types of patterns: the random clusters are generally more concentric while SOC avalanches are more varied and occasionally elongated (see, e.g., Fig. 2d).

**Figure 5:**
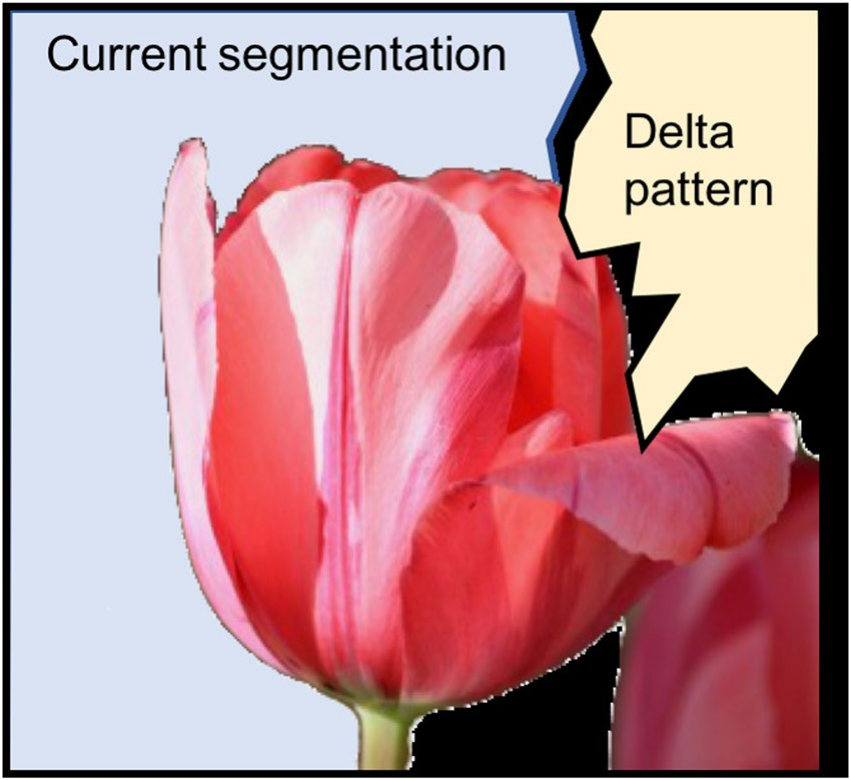
The SOC process results in delta patterns of a wide variety of sizes and shapes, increasing the probability that a delta pattern brings a segmentation out of a local minimum.

The image-segmentation experiment further provides an illustration of how SOC overcomes local minima (Fig. 5). Since the avalanches come in a wide variety of sizes and shapes (Figs 1 and 2d), it is possible that a pattern occurs that complements a current segmentation sufficiently well to overcome a local minimum. Apparently, the distribution and variability of shapes is well (maybe optimally?) balanced to allow for efficient optimization. This topic is subject to further theoretic investigation. Future work will investigate if SOC patterns are indeed optimal and which characteristics of the avalanche shape are responsible for an optimal performance.

SOC search cannot solve all NP-hard optimization problems. The optimization variables have to be linked in a graph; i.e., neighboring variables have to have some correlation (positive or negative) with each other. Moreover, there are limits on the graph structure. A fully connected graph is unsuitable because there is no locality and the path-connectedness of an avalanche would be irrelevant, eliminating the benefit over random search with random dots. For example, as we have seen for graph coloring, if we get closer to a fully connected graph, SOC search suffers. In addition, the graph structure has to be able to support SOC, e.g., the Abelian sandpile model fails to converge to SOC on line or ring graphs.

To conclude, SOC search appears remarkably adept at recovering from local minima - without any annealing scheme. Maybe this ability helps the brain from getting stuck in a thought process. Interestingly, during tonic-clonic seizures, the brain can get stuck in a repetitive loop and, as mentioned above, during seizures, criticality is apparently lost.

### Data availability

The datasets generated and analyzed during the current study are available from the corresponding author on reasonable request.
